# Inhibition of 2-AG hydrolysis alleviates posttraumatic headache attributed to mild traumatic brain injury

**DOI:** 10.1186/s10194-024-01817-z

**Published:** 2024-07-16

**Authors:** Jie Wen, Mikiei Tanaka, Yumin Zhang

**Affiliations:** Department of Anatomy, Physiology and Genetics, Uniformed Services University of the Health Sciences, 4301 Jones Bridge Road, Bethesda, MD 20814 USA

## Abstract

**Background:**

Posttraumatic headache (PTH) is a common and debilitating symptom following repetitive mild traumatic brain injury (rmTBI), and it mainly resembles a migraine-like phenotype. While modulation of the endocannabinoid system (ECS) is effective in treating TBI and various types of pain including migraine, the role of augmentation of endocannabinoids in treating PTH has not been investigated.

**Methods:**

Repetitive mild TBI was induced in male C57BL/6J mice using the non-invasive close-head impact model of engineered rotational acceleration (CHIMERA). Periorbital allodynia was assessed using von Frey filaments and determined by the “Up-Down” method. Immunofluorescence staining was employed to investigate glial cell activation and calcitonin gene-related peptide (CGRP) expression in the trigeminal ganglion (TG) and trigeminal nucleus caudalis (TNC) of the rmTBI mice. Levels of 2-arachidonoyl glycerol (2-AG), anandamide (AEA), and arachidonic acid (AA) in the TG, medulla (including TNC), and periaqueductal gray (PAG) were measured by mass spectrometry. The therapeutic effect of endocannabinoid modulation on PTH was also assessed.

**Results:**

The rmTBI mice exhibited significantly increased cephalic pain hypersensitivity compared to the sham controls. MJN110, a potent and selective inhibitor of the 2-AG hydrolytic enzyme monoacylglycerol lipase (MAGL), dose-dependently attenuated periorbital allodynia in the rmTBI animals. Administration of CGRP at 0.01 mg/kg reinstated periorbital allodynia in the rmTBI animals on days 33 and 45 post-injury but had no effect in the sham and MJN110 treatment groups. Activation of glial cells along with increased production of CGRP in the TG and TNC at 7 and 14 days post-rmTBI were attenuated by MJN110 treatment. The anti-inflammatory and anti-nociceptive effects of MJN110 were partially mediated by cannabinoid receptor activation, and the pain-suppressive effect of MJN110 was completely blocked by co-administration of DO34, an inhibitor of 2-AG synthase. The levels of 2-AG in TG, TNC and PAG were decreased in TBI animals, significantly elevated and further reduced by the selective inhibitors of 2-AG hydrolytic and synthetic enzymes, respectively.

**Conclusion:**

Enhancing endogenous levels of 2-AG appears to be an effective strategy for the treatment of PTH by attenuating pain initiation and transmission in the trigeminal pathway and facilitating descending pain inhibitory modulation.

## Background

Posttraumatic headache (PTH) is one of the most common and debilitating symptoms associated with traumatic brain injury (TBI), particularly in mild cases compared to moderate and severe types of TBI [1, 2]. PTH typically emerges within seven days post-injury, and a significant number of patients may experience persistent headache lasting for months or even years. Although classified as a secondary headache, PTH often resembles migraine and tension-type headaches [1], suggesting that it may share common and distinct mechanisms with primary headache disorders.

Enhanced nociceptive signaling in the trigeminovascular system (TGVS) and impaired descending inhibitory pain modulation likely contribute to the initiation and propagation of PTH, similar to the mechanisms observed in migraines [3]. The trigeminal ganglion (TG) receives nociceptive inputs via Aδ- and C-fibers projecting to the meninges and cerebral vasculature and transmits these signals to the brain stem and higher brain centers [4]. The release of inflammatory molecules such as nitric oxide, cytokines and prostaglandins, along with the production of neurogenic peptides like calcitonin gene-related peptide (CGRP) and pituitary adenylate cyclase-activating polypeptide (PACAP), has been reported to induce cephalic cutaneous allodynia in the PTH animal models [5, 6]. Consistently, Suppression of inflammation and neurogenic peptide production can ameliorate PTH symptoms. Although several medications such as non-steroidal anti-inflammatory drugs (NSAIDs) [7], serotonin (5-HT1) receptor agonists [8], CGRP receptor antagonists, and anti-CGRP monoclonal antibodies [9, 10] have been used for the treatment of migraine and therefore might be also suitable for PTH, they are unlikely to provide efficient pain relief in TBI patients due to the complex pathophysiology of PTH. Therefore, developing novel therapeutic agents remains an unmet clinical need.

Modulation of the endocannabinoid system (ECS) has been shown to be effective in treating inflammatory and neurodegenerative diseases, TBI, and various types of pain [11–16]. The ECS is composed of endocannabinoids, including 2-arachidonoyl glycerol (2-AG) and N-arachidonoyl ethanolamine (anandamide, AEA), cannabinoid type 1 (CB1R) and type 2 (CB2R) receptors, and enzymes for their synthesis and degradation. 2-AG is synthesized by diacylglycerol lipase (DAGL) and primarily degraded by monoacylglycerol lipase (MAGL), while AEA is mostly synthesized by N-acyl phosphatidylethanolamine phospholipase D (NAPE-PLD) and hydrolyzed by fatty acid amide hydrolase (FAAH) [17]. The ECS modulates neurotransmission in the central and peripheral nervous systems via presynaptic CB1 receptors and controls inflammation mainly through CB2 receptors [18, 19]. Augmentation of endocannabinoids is considered an ideal strategy for treating both acute and chronic pain due to their ‘on-demand’ synthesis in key regions of the nociceptive pathway, and avoidance of the psychotropic side effects associated with opioids and synthetic and plant-derived cannabinoids [20, 21].

Evidence from numerous preclinical and clinical studies highlights the antinociceptive effects of endocannabinoids in migraine [11, 22]. Patients with migraines have been found to have significant low levels of the endocannabinoid anandamide (AEA) in their cerebrospinal fluid (CSF) and serum [23, 24], supporting the hypothesis of clinical endocannabinoid deficiency in the pathogenesis of migraine [25]. In nitroglycerin-induced migraine animal model, dual inhibition of FAAH and MAGL was shown to reduce cephalic pain signals via inhibiting CGRP production and proinflammatory cytokines [12]. A recent study showed that MAGL activity in the trigeminal ganglion is significantly higher than that of FAAH [26], suggesting that controlling 2-AG metabolism might be crucial for the modulation of pain.

Despite the existence of several TBI animal models for studying PTH, each of them has notable limitations [27, 28]. The weight-drop model is commonly used but rarely produces the speed acceleration characteristic of head trauma [29]. Controlled cortical impact and fluid percussion-induced TBI animal models are also utilized in PTH studies [30, 31], but the required open surgery could damage the dura mater and thus may confound the PTH explanation. The recently developed closed-head impact model of engineered rotational acceleration (CHIMERA) may avoid these issues [32]. Unlike other TBI models, CHIMERA employs a noninvasive, surgery-free procedure to produce acceleration and deceleration head injury. It offers an advantage over the commonly used weight-drop model as the unrestrained head during impact allows for kinematic analysis of head movement. Its adjustable biomechanical input parameters enable precise replication of human TBI conditions, integrating kinematic analyses with behavioral and neuropathological outcomes. The TBI animals induced by CHIMERA has been shown to produce diffuse axonal injury, glial activation and impaired synaptic function that closely mimic human TBI neuropathology [32, 33]. These features make CHIMERA an ideal model system for investigating TBI mechanisms and for preclinical drug intervention.

Although migraine is a major headache phenotype of PTH and numerous studies have demonstrated the therapeutic potential of endocannabinoids in treating TBI and migraine, the role of endocannabinoids in the treatment of PTH has not been explored [5]. In our current study using a novel PTH mouse model induced by the CHIMERA device, we found that treatment with the selective MAGL inhibitor MJN110 [34, 35] suppressed TBI-induced cephalic pain, glial cell accumulation, and the production of the neurogenic peptide CGRP in the trigeminal system. These effects were mediated, at least in part, by cannabinoid receptor-dependent mechanisms.

## **Materials and methods**

### Materials

The MAGL inhibitor MJN110, the cannabinoid type 1 receptor (CB1R) antagonist AM281 and the type 2 receptor (CB2R) antagonist AM630 were purchased from Cayman Chemicals (Ann Arbor, MI). The DAGL inhibitor DO34, and other chemicals and reagents were purchased from Sigma (St. Louis, MO), unless stated otherwise.

### Animals

10-week-old, male C57BL/6J mice from the Jackson Laboratory (Bar Harbor, ME) were used in this study. Animal care and experimental procedures were carried out in accordance with NIH guidelines and approved by the Uniformed Services University Animal Care and Use Committee (IACUC), and the Animal Care and Use Review Office (ACUCO) from the United States Department of Defense (DoD).

### rmTBI surgery

The newly developed CHIMERA device was used to induce repetitive mild TBI (rmTBI) [32]. Following anesthesia by isoflurane (3% for induction and 2% for maintenance), mice were restrained in the supine position at an angle of approximately 32° on the CHIMERA device. The mouse head was free to move and the frontal and parietal bones lied flat over the hole in the head plate. TBI animals received a single closed-head impact on the center of the scalp in the location aligned with bregma on the skull once a day for 4 days at 0.7 J of energy using a 50-g stainless steel piston. Sham animals received an equivalent isoflurane exposure as TBI animals without receiving impact.

### Cephalic cutaneous allodynia

Mechanical allodynia was determined by testing the withdrawal response following the tactile stimulus with von Frey filaments of varying thickness. Mechanical thresholds were determined by the “Up-Down” methods [36, 37]. Mice were placed inside plastic red tube restrainers and allowed to acclimate for at least 5 min once a day for two days before testing. A series of von Frey filaments (Stoelting, Wood Dale, IL) with logarithmically incremental stiffness ranging from 2.44 to 4.31 (0.16–6.0 g) were applied to the middle periorbital region over the rostral portion of the eyes. To elicit a positive response, the filament needs to make a firm perpendicular contact to the skin and causes a slight bend. A positive response is recorded when the mouse vigorously strokes its face with the forepaw, head withdrawal from the stimulus, or head shaking by three repeated stimuli [37].

### Drug treatment

All drugs were dissolved in DMSO-cremophor-saline (1:1:18), which was used as a vehicle control. Animals were randomly assigned to sham, rmTBI and MJN110 treatment groups. Drugs were given intraperitoneally (i.p.) 1 h after each impact and then continued for 3 additional days (7 days in total). To determine the cannabinoid receptor dependency, MJN110 (2.5 mg/kg) was co-administered with the CB1R antagonist AM281 (3 mg/kg, i.p.) or the CB2R antagonist AM630 (3 mg/kg, i.p.), respectively.

### Immunohistochemistry (IHC)

Following euthanasia with ketamine and xylazine (90 mg ketamine/10 mg xylazine per ml, 10 µl/g body weight, i.p), animals were intracardially perfused with ice cold 1x PBS and then 4% paraformaldehyde in the same buffer. Trigeminal ganglion and brain were dissected out and kept in 4% paraformaldehyde at 4 °C overnight. Tissues were washed with 1x PBS twice and transferred into 20% sucrose in PBS at 4 °C until sinking to the bottom. The precipitated tissues were embedded in Tissue Tek OCT and stored at − 80 °C. Brain stem and trigeminal ganglion were sectioned at 25 μm using cryostat (Leica model CM1950, Bannockburn, IL) and mounted onto Superfrost Plus slides for immunoassay. Anti-rat Iba1 (1:300; Abcam, Cambridge, MA), anti-mouse GFAP (1:500; Cell Signaling, Danvers, MA) and anti-mouse CGRP (1:100; Santa Cruz, CA) primary antibodies were used for IHC. Briefly, the slides were washed with PBS twice, and then incubated in PBS blocking buffer containing 5% donkey serum and 0.3% Triton X-100 for 30 min at room temperature, followed by primary antibodies added in the same buffer overnight. The slides were then washed with PBS containing 0.2% Triton X-100 three times and incubated for 1 h with Alexa Fluor 488, Alexa Fluor 594 or Alexa Fluor 655 conjugated donkey anti-rabbit, mouse or rat secondary antibodies (1:750; Thermo Fisher Scientific, Waltham, MA). The slides were again washed with PBS and covered with Fluoroshield mounting medium with DAPI. Images were obtained in a minimum of 5–7 serial sections from the trigeminal ganglion or medulla of the same animal with a fluorescence microscope (Nikon Eclipse TE-2000 U). The Iba1, GFAP and CGRP immunoreactive cells were counted using ImageJ software. Regions of interest (ROI) were selected, and cell counts were initiated using the cell counter plugin. Iba1 immunoreactive macrophages in TG were also counted manually to exclude the false positive immunoreactivity including NeuN positive cells. The average number of positively stained cells within the ROI from at least four serial sections in each animal was calculated and expressed as the number of positively stained cells per square millimeter (cells/mm²).

### qRT-PCR

On day 7 post-rmTBI, mice were euthanized and the whole brain and trigeminal ganglion were dissected out and quickly frozen. For trigeminal nucleus caudalis (TNC), frozen brain was coronally sliced at 200 µm thickness − 8.2 mm to -7.0 mm from bregma using a cryostat. TNC was cut off from these frozen slices. The tissues were homogenized with 0.5 ml of TRIzol. Total RNA was isolated by a combination method of TRIzol phase separation and spin column purification with on-column DNase I digestion (ZymoResearch). Complementary DNA was prepared with MAXIMA cDNA synthesis kit followed by qRT-PCR using SYBR green Powerup master mix (Thermo Fisher Scientific) to assess the relative expression levels of the genes of interest by normalizing with that of GAPDH. The primer sequences used include Clr: forward, 5’-atctcagcagagtcggaagaa-3’, reverse, 5’-caggtcctattgcaagtaaaggc-3’, Ramp1: forward, 5’-gagactattgggaagacgctatg-3’, reverse, 5’-ctcctccagaccaccagtg-3’, and GAPDH: forward, 5’- aggtcggtgtgaacggatttg-3’ and reverse, 5’- tgtagaccatgtagttgaggtca-3’. The relative levels of gene expression were determined by the 2^−ΔΔCt^ method.

### Mass spectrometry

The tissue was homogenized with 40 µl of 0.02% trifluoroacetic acid, 60 µl of acetonitrile including 2-AG-d5 (40 ng), AA-d11 (400 ng), and AEA-d4 (20 pg) (Cayman Chemicals) using a Potter homogenizer at 4 °C. The homogenate was dissolved completely in 1 ml acetonitrile by vortex and kept at 4 °C overnight followed by centrifugation at 1,000 g for 10 min to remove the debris. The supernatant was evaporated under nitrogen gas streaming in a water bath (approx. 35 °C) and the lipid was resuspended with 100 µl of acetonitrile and stored at − 80 °C until use. Quantification of 2-AG, AA, and AEA using liquid chromatography coupled with mass spectrometry (LC/MS) was performed as we previously described [15].

### Statistical analysis

Data were analyzed with analysis of variance (ANOVA). One way ANOVA was used to compare the data obtained from PCR, mass spectrometry, cell number and protein intensity of images among the different experimental groups, while the two-way repeated measures ANOVA was used for comparison of multiple data sets in behavioral tests. Tukey-Kramer post hoc analysis was used for comparing the different treatment groups. Results were presented as mean ± standard error of the mean (SEM). A significant difference was determined as *p* < 0.05.

## Results

### Tactile hypersensitivity following CHIMERA induced rmTBI was attenuated by the 2-AG hydrolysis inhibitor MJN110

**Fig. 1:**
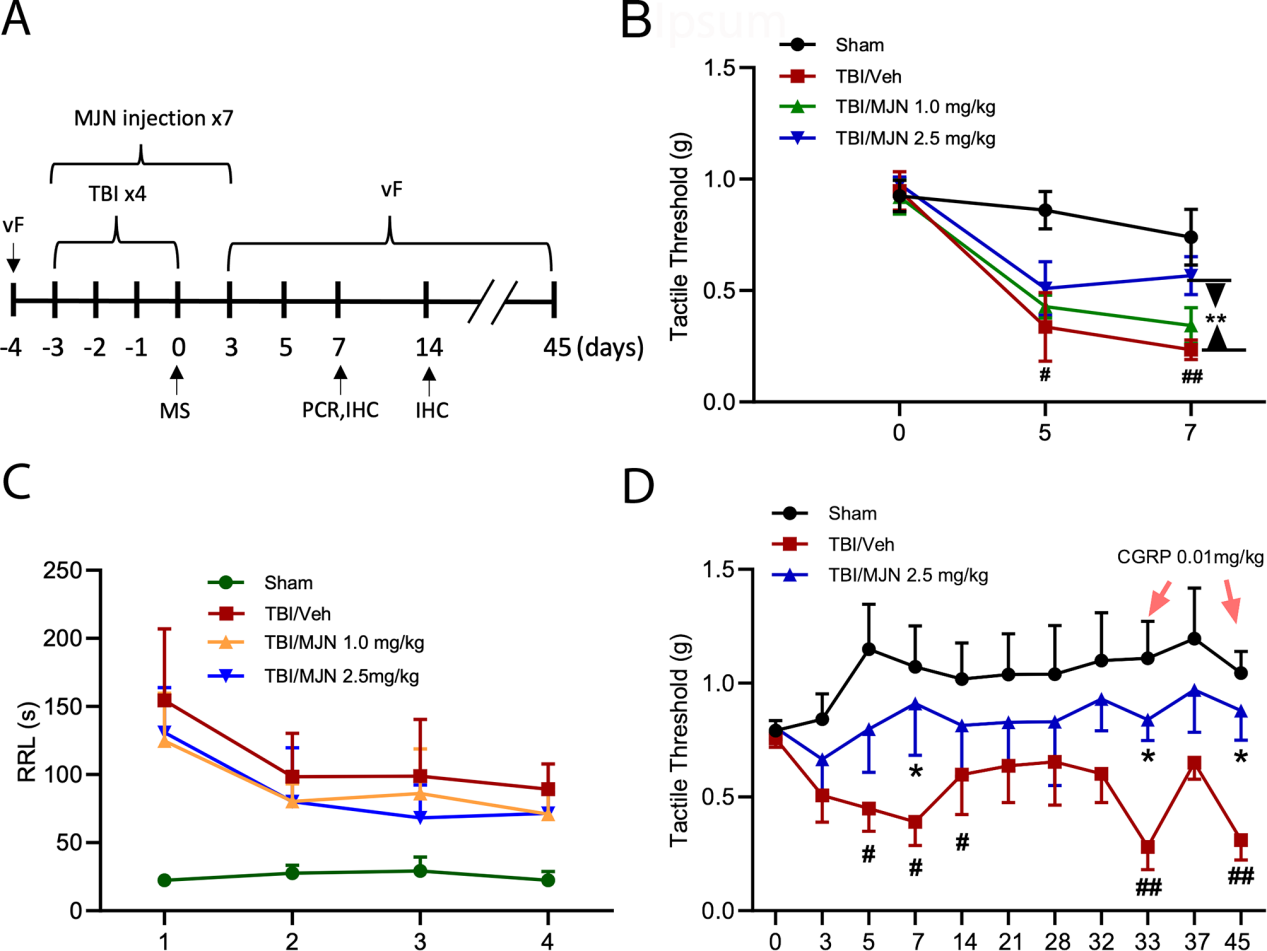
MJN110 dose dependently alleviated periorbital allodynia and reduced persistent pain sensitivity in the mTBI mice. The timeline and various experimental procedures were illustrated in Fig. 1A. von Frey (vF) test, mass spectrometry (MS), PCR and immunohistochemistry (IHC) were performed at the times indicated (**A**). On days 5 and 7 post-injury, the TBI/vehicle group showed a significantly reduced periorbital tactile threshold compared to the sham group (**B**). Animals treated with low dose of MJN110 (MJN, 1 mg/kg) displayed similar mechanical response as the TBI/vehicle animals. At 2.5 mg/kg, MJN increased mechanical thresholds on day 5 and day 7 post-injury compared to the TBI/vehicle group. **, *p* < 0.01 was obtained when the MJN treated group was compared to the TBI/vehicle group (*n* = 15/group). #, *p* < 0.05, ##, *p* < 0.01 was obtained when the TBI/vehicle group was compared to the sham group (*n* = 15/group). The righting reflex latency (RRL) was recorded after each impact (**C**). Compared to the sham group, both TBI/vehicle and MJN treatment groups showed longer RRL. No difference was found between the TBI/vehicle and MJN treated groups. CGRP (0.01 mg/kg) was given on day 33 and day 45 post-injury to the sham, TBI/vehicle and MJN treated groups and periorbital allodynia was assessed (**D**). There was no pain sensitivity change in both sham and the MJN treated groups, but the TBI/vehicle group exhibited significantly increased pain sensitivity compared to the sham and the MJN treatment group. *, *p* < 0.05 was obtained when the MJN treated group was compared to the TBI/vehicle group (*n* = 12/group). #, *p *< 0.05, and ##, *p* < 0.01 were obtained when the TBI/vehicle group was compared to sham group (*n* = 12/group)

Repetitive head impacts, drug treatments and various experimental procedures were described in the experimental diagram (Fig. 1A). To determine if animals develop periorbital tactile hypersensitivity following rmTBI induced by a CHIMERA device, von Frey filaments were utilized to stimulate the periorbital region and the changes in tactile sensitivity were evaluated by the up-down method [38] at 5 and 7 days post-injury. Mice with rmTBI showed a significant reduction in periorbital tactile threshold compared to the baseline and the sham group on day 5 and day 7 post-injury (Fig. 1B). Despite no significant changes were observed in the 1.0 mg/kg MJN110 group, treatment with MJN110 at 2.5 mg/kg led to a significant increase in the tactile threshold from 0.23 g in the TBI/vehicle group to 0.56 g at 7 days post-injury (Fig. 1B). Administration of MJN110 at 2.5 mg/kg to the sham group did not alter the tactile threshold (data not shown). Notably, TBI animals had significantly increased righting reflex, but the use of MJN110 at both doses did not improve the righting reflex latency after each impact (Fig. 1C), suggesting that MJN110 treatment had no effect on the loss of consciousness caused by the direct TBI impact.

Several studies have shown that rmTBI mice possess latent pain hypersensitivity to migraine inducing agents, such as nitroglycerin and CGRP [39–41]. To determine the role of MJN110 on persistent pain hypersensitivity, rmTBI animals were continuously measured by von Frey filaments until 45 days post-injury. As shown in Fig. 1D, the tactile threshold in the periorbital region was significantly reduced at 5, 7 and 14 days post-injury and remained at low threshold for at least a month although no significant difference was found between the rmTBI and sham control groups at any late time point. Treatment with MJN110 at 2.5 mg/kg increased the tactile threshold at all time points, but the significant difference between the drug treatment group and the TBI/vehicle group was only observed at 7 days post-injury. To determine whether rmTBI animals exhibited latent pain hypersensitivity to the neurogenic CGRP peptide, all animals received an intraperitoneal (i.p.) injection of CGRP at 0.01 mg/kg on days 33 and 45 post-rmTBI. Consistent with previous findings [41], the rmTBI/vehicle group showed exaggerated allodynia, with the lowest tactile thresholds of 0.27 g on day 33 and 0.31 g on day 45, assessed one hour after CGRP administration. In contrast, CGRP injection did not alter tactile thresholds in the sham group and the MJN110 treatment group, suggesting that MJN110 treatment prevented latent pain hypersensitization or pain chronification in the rmTBI animals.

### Accumulation of microglia/macrophages and astrocytes in the trigeminal ganglia (TG) and trigeminal nucleus caudalis (TNC) of the rmTBI animals was ameliorated by MJN110

**Fig. 2:**
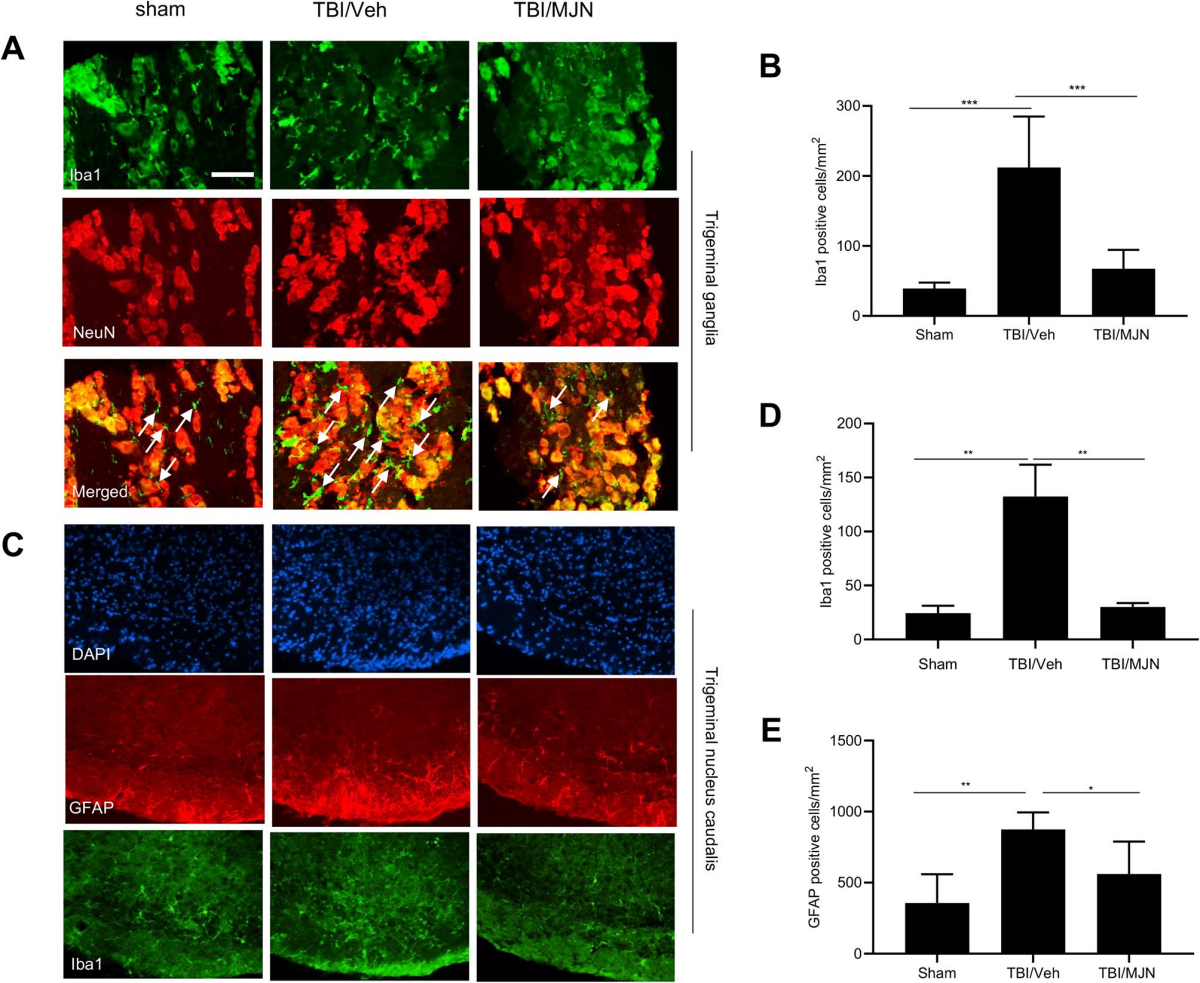
The increased glial cells accumulation in the trigeminal ganglion (TG) and the trigeminal nucleus caudalis (TNC) of the mTBI mice was attenuated by MJN110 treatment. On day 7 post-injury, the Iba1-positive macrophages/microglia cells significantly increased in the TG (**A**; arrows in the merged images point to the representative macrophages) and TNC (**C**) of the mTBI mice, which were remarkably reduced by MJN treatment. Quantification showed that there was 4–5 folds increase in the Iba1 positive cells in the TBI/vehicle group compared to the sham group in both TG and TNC, and the increase was almost completely blocked by MJN treatment (**B**, **D**). ***, *p* < 0.001, **, *p* < 0.01 was obtained when the MJN treated group compared to the TBI/vehicle group. GFAP positively stained cells in the TNC significantly increased compared to that in the sham group, and was remarkably reduced by the MJN treatment (C, E), *, *p* < 0.05 was obtained when the MJN treatment group was compared to the TBI/vehicle group (*n* = 10/group). Scale bar = 50 μm

At 7 days post-rmTBI, the expression of macrophages in TG was examined based on Iba1 immunostaining. The Iba1 immunoreactive macrophages in TG of the TBI/vehicle group were increased compared to the sham group (Fig. 2A). Iba1 positively stained cells were found at close proximity to neurons, and MJN110 treatment attenuated the expression of Iba1 (Fig. 2A). Quantification of the number of positive Iba1 cells showed a five-fold increase in the rmTBI/vehicle group compared to the sham and MJN110 treatment groups (Fig. 2B). The number of Iba1 positive cells increased from 39 ± 3 cells/mm^2^ in the sham group to 212 ± 20 cells/mm^2^ in the rmTBI/vehicle group, which was significantly reduced by the MJN110 treatment (67 ± 10 cells/mm^2^; Fig. 2B).

The expression of GFAP immunoreactive astrocytes and Iba1 positive microglia was also examined in the TNC region (Fig. 2C). The rmTBI animals had a robust GFAP and Iba1 immunoreactivity in the TNC compared to the sham group. Treatment with MJN110 dramatically reduced the expression of the GFAP and Iba1 (Fig. 2C). Quantification indicated that the Iba1 and GFAP positive cells were 132 ± 15 and 873 ± 54 cells/mm^2^ in the rmTBI/vehicle group, which were significantly greater than 25 ± 4 and 357 ± 90 cells/mm^2^ in the sham group. The increased Iba1 and GFAP positive cells in the rmTBI group were brought down to 30 ± 2 and 561 ± 94 cells/mm^2^ by the MJN110 treatment (Fig. 2D, E).

### MJN110 treatment attenuated CGRP expression in the rmTBI mouse TG and TNC

**Fig. 3:**
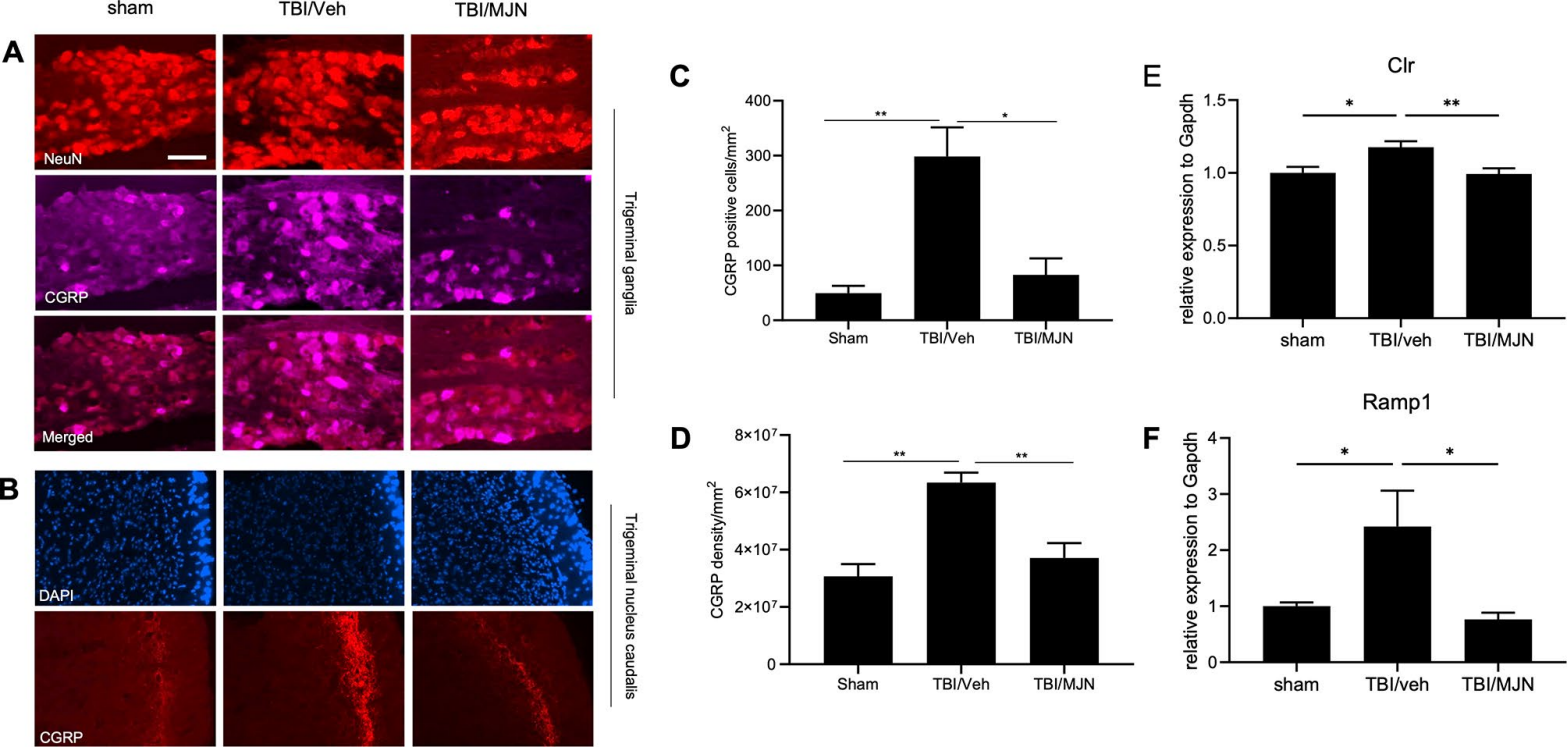
Treatment with MJN110 inhibited the expression of calcitonin gene related peptides (CGRP) in TG and TNC of mTBI mice. On day 7 post-injury, the CGRP positive immunostaining increased in the TG and TNC of mTBI mice compared to the sham group and decreased by the MJN treatment (**A**, **C**). The number of positive CGRP cells in the TG of mTBI mice was significantly higher than that in the sham group and greatly reduced by MJN (**B**). The same phenomenon was observed in the TNC of mTBI mice (**D**). *, *p* < 0.05, and **, *p* < 0.01 were obtained when the MJN treatment group compared to the TBI/vehicle group (*n* = 10). The mRNA levels of the calcitonin-receptor like receptor (*Clr*) (**E**) and the receptor activity modifying protein 1 (*Ramp1*) (**F**) in the TG were also increased in the TBI/vehicle group and suppressed in the MJN treatment group (*n* = 12/group). Scale bar = 50 μm

In the TG, the expression of CGRP was mostly found in neurons and increased at 7 days post-injury. Treatment with MJN110 had an inhibitory effect on the increased CGRP expression (Fig. 3A). In the TNC, the expression of CGRP was seen in the superficial laminae, and was elevated in the rmTBI/vehicle group and reduced in the MJN110 treatment group (Fig. 3B). Quantification of CGRP showed that rmTBI injury resulted in a 5-fold increase in the TG (Fig. 3C) and a 2-fold increase in the TNC (Fig. 3D) compared to the sham and the MJN110 treatment groups. The expression of CGRP receptors, the calcitonin receptor-like receptor (*Clr*) (Fig. 3E) and the receptor activity modifying protein 1 (*Ramp1*) (Fig. 3F) in the TG was also increased in the rmTBI/vehicle group and suppressed in the MJN110 treatment group.

### The suppressive effect of MJN110 on TBI induced periorbital allodynia was mediated through the cannabinoid signaling pathway

**Fig. 4:**
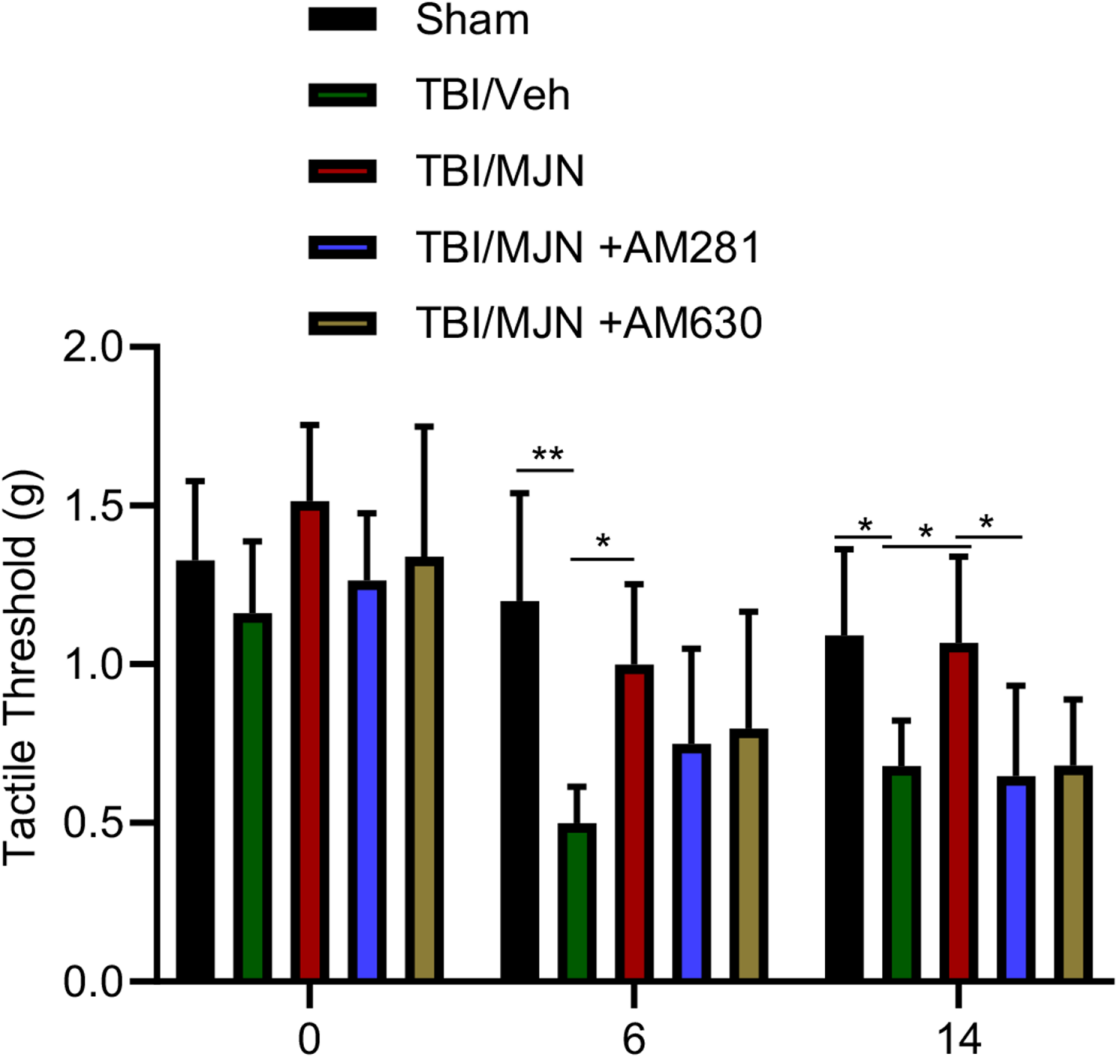
The anti-nociceptive effect of MJN110 in the mTBI mice was dependent on the cannabinoid receptor activation. MJN was co-administered with either the CB1R antagonist AM281 (3 mg/kg) or the CB2R receptor antagonist AM630 (3 mg/kg) in the mTBI mice once a day for a total of 7 days. Treatment with MJN showed a significant reduction of allodynia at 6 and 14 days post-TBI. Combination of MJN with AM630 partially reversed MJN110 mediated anti-allodynic effect on days 6 and 14; while combination of MJN with AM281 resulted in significantly decreased tactile threshold compared to the MJN alone treated group on day 14. *p < 0.05 and **p < 0.01 (*n* = 15/group)

To determine if the therapeutic effect of MJN110 on PTH relies on the cannabinoid signaling pathway, TBI mice were given MJN110 together with the CB1R antagonist AM281 or the CB2R antagonist AM630. Periorbital cutaneous allodynia was examined at 6 and 14 days post-TBI. A significantly reduced tactile threshold was seen in the rmTBI/vehicle group compared to the sham group at both time points (Fig. 4). Administration of AM281 and AM630 partially reversed the improved periorbital allodynia (increased tactile threshold) observed in the MJN110 alone treatment group. On day 6, TBI animals treated with MJN110 with the CB1R or the CB2R antagonist showed a moderately reduced tactile threshold compared to the MJN110 treatment alone. On day 14 post-rmTBI, animals treated with MJN110 had an increased tactile threshold of 1.06 g, which is significantly higher than of the rmTBI/vehicle group (0.67 g, *p* < 0.05). Co-administration of AM281 or AM630 together with MJN110 rendered the tactile thresholds (0.64 g and 0.68 g in the MJN110 + AM281 and MJN110 + AM630 groups, respectively) back to the level observed in the TBI/vehicle group (0.67 g). Despite both CB1 and CB2 receptors seemed to mediate the therapeutic effect of MJN110, a significant difference was only observed in the MJN110 and AM281 treatment group at 14 days post-TBI.

### The anti-inflammatory effect of MJN110 in TG and TNC of the TBI mice was reversed by the cannabinoid receptor antagonists

**Fig. 5:**
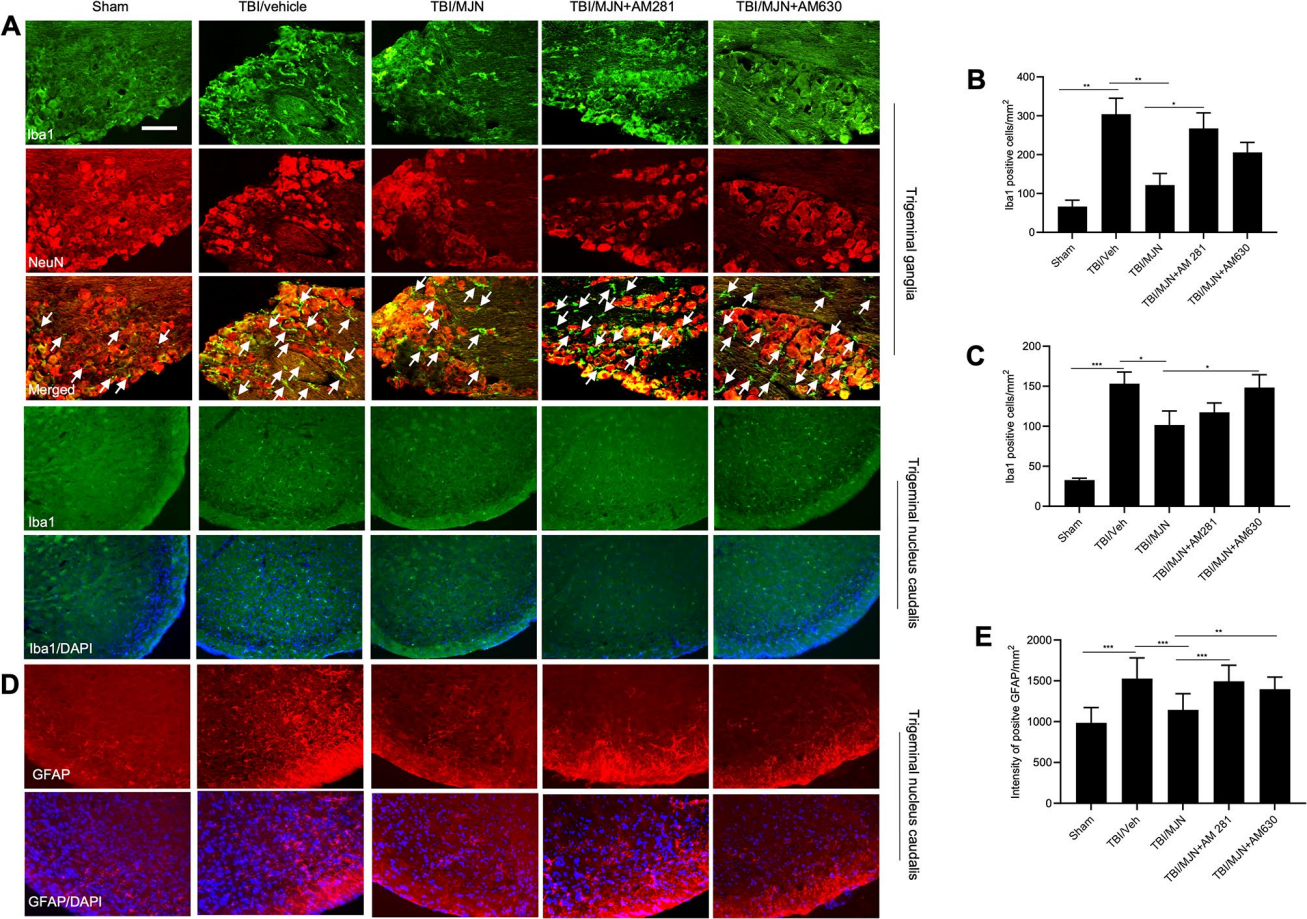
The elevated astrocytes and microglia/macrophages accumulation in TG and TNC of mTBI mice was attenuated by MJN110 in a cannabinoid receptor dependent manner. On day 14 post-injury, the Iba1-positive immunostaining was significantly increased in the TG and TNC (**A**; arrows in the merged images point to macrophages) of the mTBI mice. MJN treatment remarkably reduced positive Iba1 cells in both regions (**B**, **C**). Co-administration of AM281 significantly reversed the MJN inhibitory effect on macrophages accumulation in the TG (**B**), but not in the TNC (**C**). Conversely, addition of AM630 significantly reversed the MJN effect in TNC (**C**), but not in TG (**B**). Both CB1 and CB2 receptor antagonists reversed MJN effect on GFAP accumulation in the TNC (**D**, **E**). *, *p* < 0.05, **, *p* < 0.01, and ***, *p* < 0.001 (*n* = 8/group). Scale bar = 50 μm

rmTBI mice were given MJN110 with or without the cannabinoid receptor antagonists for 7 days and then subjected to transcardial perfusion on day 14 post-injury. Compared to the sham group, there were increased Iba1 immunopositive microglia/macrophages in the TG and TNC of the TBI animals, and the increased immunoreactivity was greatly reduced by the MJN110 treatment (Fig. 5A). In the TG, the reduced expression of Iba1 positive macrophages by MJN110 was reversed by co-administration of the CB1 and CB2 receptor antagonists, with a significant difference observed in the MJN110 and AM281 treatment group (Fig. 5B). In the TNC, the MJN110 inhibitory effect on microglia activation was significantly reduced by the CB2R antagonist (Fig. 5C). Furthermore, we found that treatment with MJN110 suppressed the increased accumulation of GFAP and the inhibitory effect was reversed by addition of AM281 or AM630 (Fig. 5D). Quantification of GFAP fluorescence showed that the intensity of positive GFAP staining in animals treated with the combination of MJN110 and the CB1 or the CB2 receptor antagonist was significantly greater than that in the group of MJN110 treatment alone, and it was almost back to the level of the TBI/vehicle group (Fig. 5E).

### Attenuation of the CGRP expression by MJN110 in the mouse TG and TNC was regulated through cannabinoid receptors

**Fig. 6:**
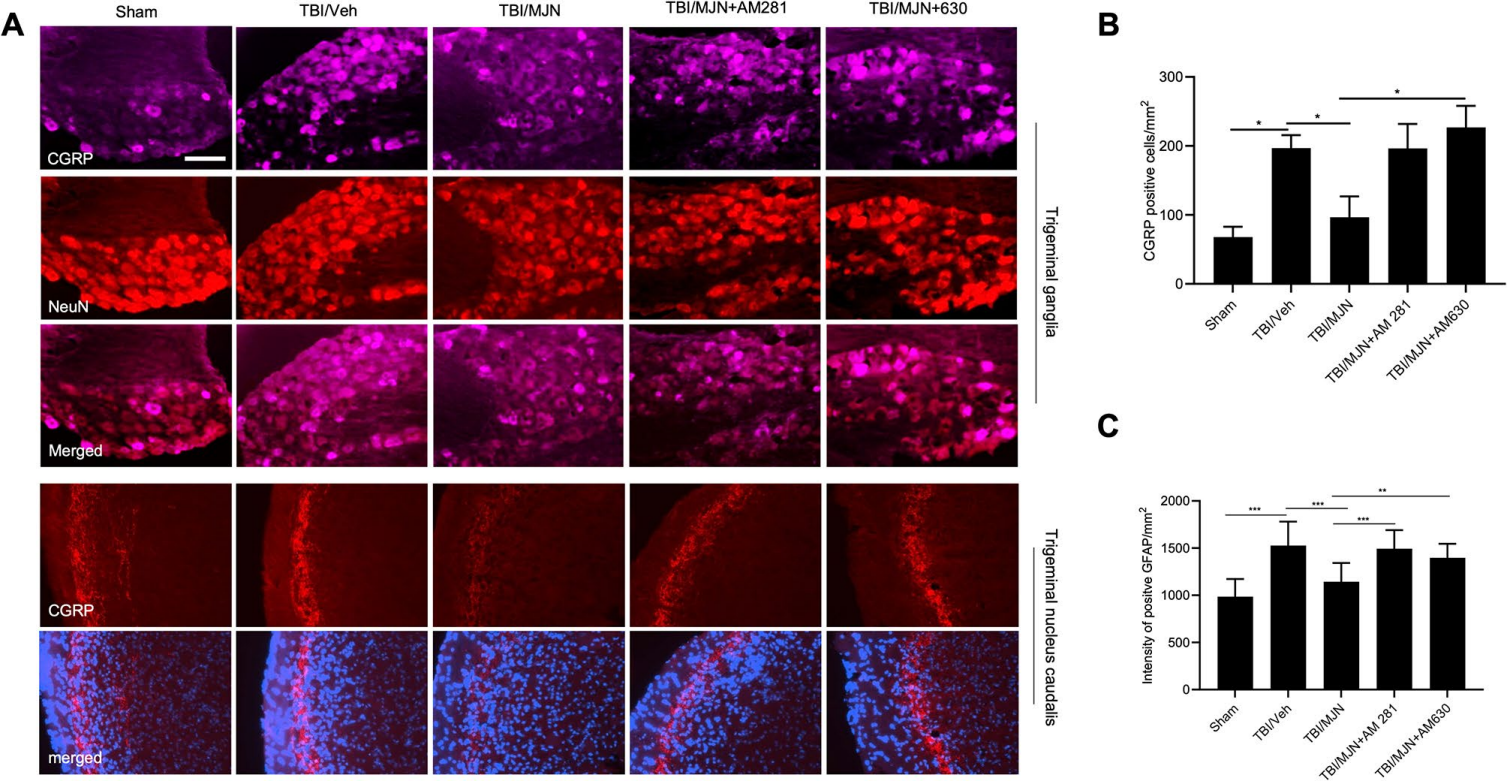
Attenuation of MJN110 on the expression of CGRP in both TG and TNC of the mTBI mice was regulated by cannabinoid signaling pathways. On day 14 post-injury, the CGRP-positive immunostaining was significantly increased in the TG and TNC of the mTBI mice, and MJN treatment remarkably attenuated CGRP immunostaining in both regions (A). In the TG of mTBI mice, The CB2R antagonist AM630 significantly counteracted the downregulation of CGRP by MJN (B). In the TNC of mTBI mice, animals treated with MJN and the CB1R antagonist AM281 had significantly increased CGRP positive immunostaining compared to the MJN alone treated group (C). *, *p* < 0.05, **, *p* < 0.01, and ***, *p* < 0.001 (*n* = 8/group). Scale bar = 50 μm

CGRP expression was also examined in the TG and TNC by immunohistochemistry. The CGRP expression in the TG and TNC was significantly elevated at 14 days post-rmTBI and attenuated by MJN110 treatment. Administration of the CB1R or CB2R antagonist together with MJN110 reversed the enhanced CGRP immunoreactivity (Fig. 6A). Quantification of the CGRP positive cells in the TG indicated that rmTBI animals treated with MJN110 and AM630 had significantly increased number of CGRP positive cells compared to the MJN110 alone treatment group (Fig. 6B). In the TNC region, the inhibitory effect of MJN110 on CGRP expression was mainly mediated by the CB1 receptor activation (Fig. 6C).

### The antinociceptive effect of MJN110 was blocked by inhibition of the 2-AG synthesis

**Fig. 7:**
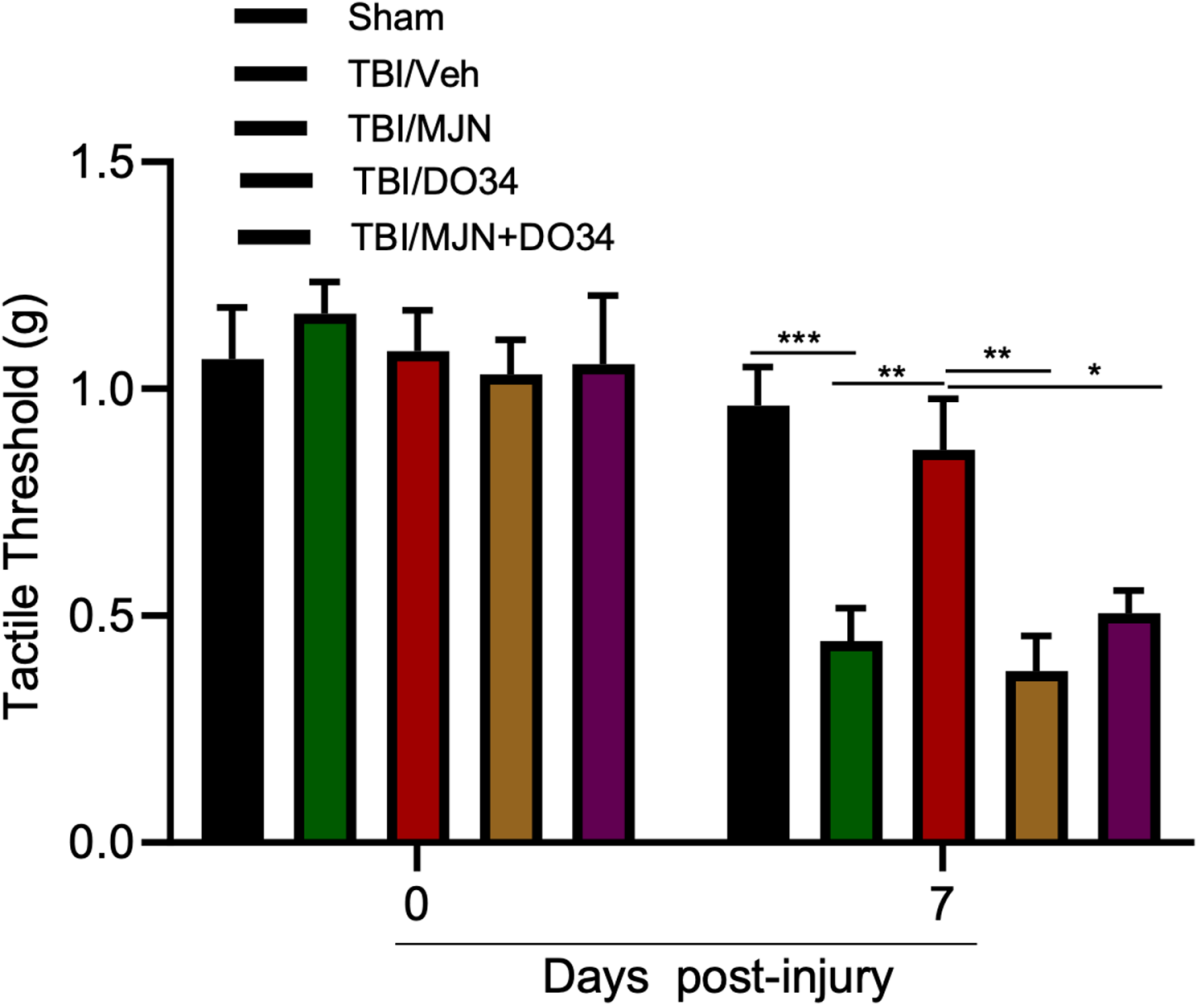
The 2-AG synthetic enzyme DAGL inhibitor DO34 blocked MJN110 mediated anti-allodynic effect in the mTBI mice. On day 7 post-TBI, MJN treatment had a significantly reduced periorbital cutaneous hypersensitivity compared to the TBI/vehicle group. DO34 treated mTBI mice behaved similar to the TBI/vehicle group. Administration of DO34 together with MJN showed similar periorbital allodynia to the TBI/vehicle group, and it significantly reversed the antinociceptive effect of the treatment of MJN alone. *, *p* < 0.05, **, *p* < 0.01, and ***, *p* < 0.001 (*n* = 10/group)

To further determine if the therapeutic effect of MJN110 is dependent upon the increased levels of 2-AG, the rmTBI animals were administered DO34 (30 mg/kg, i.p.), a selective inhibitor of the 2-AG synthetic enzymes DAGLα and DAGLβ [42], either alone or in combination with MJN110. The antinociceptive effect of MJN110 was significantly reduced by co-administration of DO34, and treatment with DO34 did not affect the tactile threshold in the rmTBI animals (Fig. 7).

**Fig. 8:**
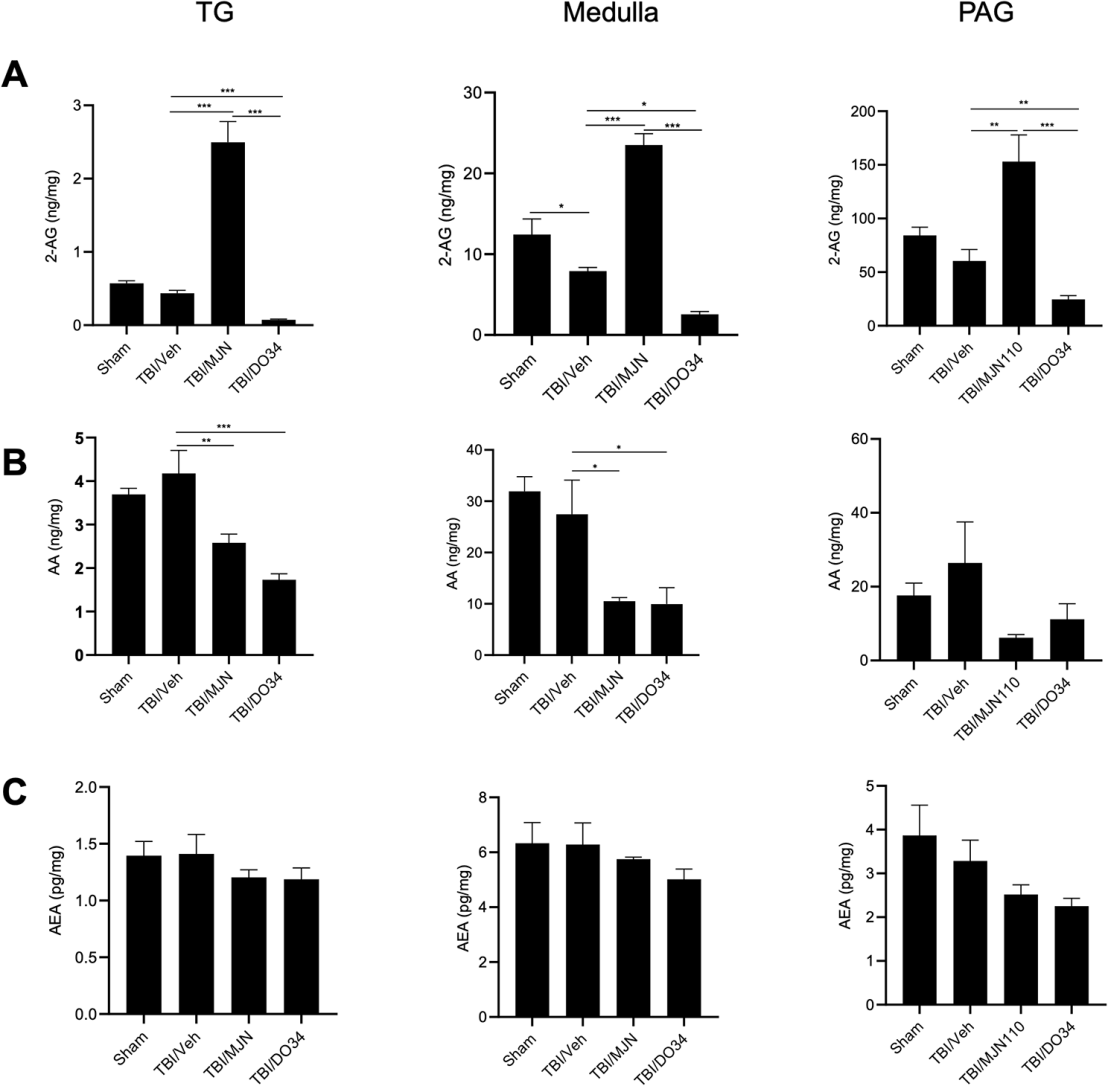
MJN110 and DO34 had opposite effects on the levels of 2-AG in TG, medulla and PAG. On day 7 post-TBI, fresh tissues from TG, medulla and PAG regions were subject to mass spectrometry to measure the 2-AG, AA and AEA contents. TBI animals treated with MJN showed highest 2-AG levels compared with the TBI/vehicle and the DO34 treatment groups in the TG, medulla and PAG regions (**A**). MJN and DO34 treatment significantly reduced AA in TG and medulla (**B**). The levels of AEA were not significantly altered in all the experimental groups (**C**). *, *p* < 0.05, **, *p* < 0.01, and ***, *p* < 0.001 (*n* = 10/group)

To determine if TBI and drug treatments altered the endocannabinoid levels in several regions associated with headache, TG, medulla containing TNC and PAG were isolated for the quantification of 2-AG, AEA and arachidonic acid (AA) using mass spectrometry. Repetitive TBI injury for 4 days resulted in a 20–40% reduction of 2-AG in all the regions examined, and a significant difference was observed in medulla (Fig. 8A). The 2-AG levels in these regions were significantly increased by MJN110, and further reduced by DO34 when compared to the TBI/vehicle group (Fig. 8A). The reduction of AA by MJN110 and DO34 was also found in the TG, medulla and PAG, supporting that 2-AG is a major substrate for AA production in these regions (Fig. 8B). On the other hand, the levels of AEA were not significantly altered in all the experimental groups, suggesting that MJN110 selectively affects the 2-AG, but not the AEA metabolism (Fig. 8C).

## Discussion

This study employed a surgery-free and non-invasive CHIMERA mouse model to induce headache associated with rmTBI. Systematic treatment with MJN110 to elevate the endogenous levels of 2-AG significantly attenuated periorbital allodynia, glial cells activation and CGRP production in the trigeminal system through CB1and CB2 cannabinoid receptor dependent mechanisms.

Numerous studies have demonstrated the pivotal role of CGRP in migraine initiation and susceptibility. Elevated CGRP level was observed in TG and TNC in a migraine model induced by electrical stimulation of the dura mater [43]. Cephalic allodynia and altered light sensitivity were also found by repeated CGRP injections [44], or intraganglionic CGRP injection [45]. Conversely, the use of a monoclonal antibody against CGRP ameliorated orofacial hyperalgesia in a migraine model [10]. The increased expression and production of CGRP in TG and TNC were also reported in both weight drop [6] and the controlled cortical impact-induced TBI animal models [46]. In this study, we found that CGRP production and the mRNA expression of its main receptor components, Clr and Ramp1, were significantly increased in TG and TNC at 1–2 weeks post-injury, and injection of CGRP at subthreshold dose provoked cephalic cutaneous hypersensitivity in TBI animals at 33 and 45 days post-injury when the initial or acute allodynia had subsided. This result is consistent with the findings from a preclinical TBI mouse model [39] and clinical studies in PTH patients [47, 48]. These results suggest that the trigeminal system is chronically sensitized after rmTBI and that sustained CGRP signaling could be one of the pathogenic mechanisms of PTH.

Many preclinical and clinical studies have suggested that migraine is a major phenotype of PTH [49]. In migraine models, the release of pro-inflammatory molecules can directly activate meningeal peripheral nerve endings, and enable them highly susceptible to chemical and mechanical stimuli [50]. Activation of glial cells in both the central and peripheral nervous systems, including satellite glial cells, plays a crucial role in the initiation and maintenance of chronic pain [51]. Microglia and macrophages are the predominant sources of pro-inflammatory cytokines and prostaglandins [52, 53]. During pain development, the pro-inflammatory mediators can directly sensitize nociceptors [54, 55]. In our current PTH mouse model, we observed a significant increase in microglia/macrophages, and astrocytes in the trigeminal system at 7 and 14 days post-injury. Pro-inflammatory cytokines and other inflammatory mediators from satellite glial cells have been shown to increase neuronal excitability and trigger the release of CGRP [56, 57]. The release of CGRP can in turn activate the CGRP receptor expressed in the satellite glial cells [58, 59]. Thus, the bidirectional neuron-glia signaling via CGRP and inflammatory mediators could further promote pain transmission in the TGVS [60]. CGRP is produced in a subset of TG neurons, which innervate the meninges and TNC [61]. The CGRP receptors, CLR and RAMP1, are expressed in A-δ fibers of TG neurons, that are located near the C-fiber nerve endings [62, 63]. Thus, the release of CGRP from C-fiber terminals can sensitize adjacent A-δ fibers to initiate pain [62]. Electrophysiological studies have also demonstrated that activation of TNC neurons by A-δ fiber stimulation promotes nociceptive neurotransmission between the TG and secondary neurons in the TNC [63].

We have previously demonstrated that inhibition of 2-AG hydrolysis with MJN110 reduces neuroinflammation in the cerebral cortex and hippocampus of the rmTBI mouse [15]. In our current study, we observed a significant reduction in Iba1 and GFAP positive cells in both TG and TNC of MJN110-treated animals. MJN110 treatment also significantly reduced the levels of AA, the precursor of proinflammatory prostaglandins and leukotrienes in TGVS-associated regions. Several studies have suggested a causal role for prostaglandin E2 (PGE2) in the pathogenesis of migraines [56, 57]. Therefore, the reduction in PGE2, due to the reduced availability of AA, is likely one of the therapeutic mechanisms of MJN110. We also found that MJN110 treatment downregulated the expression of CGRP, Clr and Ramp1 in the TG, suggesting that the therapeutic effect of MJN110 might be due to its suppression of CGRP induced nociceptive signaling. Furthermore, we observed that the TBI mice, but not sham and the MJN110 treated mice, regained cephalic hypersensitivity following CGRP administration at 33 and 45 days post-injury. These results suggest that MJN110 treatment may have both acute and prolonged therapeutic effects.

Endocannabinoids interact primarily with two Gi/o protein-coupled CB1 and CB2 cannabinoid receptors [17]. CB1 receptors are highly expressed in neurons of the central nervous system, as well as sensory neurons in the dorsal root ganglion and the TG [64]. Activation of CB1R can therefore modulate neurotransmission in both central and peripheral nerve tissues. Multiple ion channels involved in pain pathways are also regulated by CB1R activation. It has been reported that CB1R activation can suppress the activity of voltage-gated calcium channels (VGCC) and the release of CGRP [65]. Additionally, CB1R-mediated opening of potassium channels decreases excitability and reduces nociceptive spiking [66]. Studies have also shown that CB1R activation reduces neuronal sensitization by lowering cAMP and PKA levels [67]. On the other hand, the CB2 receptors are primarily found in inflammatory and immune cells [17], and their activation has been showed to attenuate neuroinflammation and neurotoxicity in TBI [47, 48, 68]. Consistently, TBI severity is exacerbated in the CB2R knockout mice [69]. In line with these findings, we found that activation of both CB1 and CB2 receptors is required for the antinociceptive effect of endogenous 2-AG. Activation of the CB1 receptors appears to be crucial for reducing the pain response, as TBI mice treated with MJN110 and the CB1R antagonist, but not the CB2R antagonist exhibited a significantly reduced mechanical threshold compared to the MJN110 alone treatment group. Since the inhibitory effects of MJN110 on glial cell accumulation and CGRP expression were reversed by the CB2R antagonist, the effects of MJN110 are likely mediated by both CB1 and CB2 cannabinoid receptors.

In addition to acting on the canonical CB1 and CB2 receptors, endocannabinoids can interact with several noncanonical cannabinoid receptors, including G protein-coupled receptors (GPR18 and GPR55), peroxisome proliferator-activated receptors (PPARs), and transient receptor potentials (TRPs) [70, 71]. It has been reported that anandamide (AEA) can activate the transient receptor potential vanilloid receptor (TRPV1) to trigger the release of CGRP and promote nociceptive signaling [72]. In the TGVS, many pain-related ion channels are located in the meningeal afferents and can be targeted by 2-AG and AEA [73, 74]. Since the activity of most ion channels depends on membrane lipids such as phosphatidylinositol 4,5-bisphosphate (PIP2) and specific fatty acids [75, 76], the endocannabinoid-metabolized lipid profile allows them to modulate mechanosensitive ion channels through noncanonical lipid signaling. Therefore, both canonical and noncanonical pathways are likely involved in endocannabinoid-mediated pain modulation.

Clinical endocannabinoid deficiency has long been hypothesized to cause migraine due to insufficient endocannabinoid tone [25, 77]. Consistently, the levels of 2-AG were recently reported to be lower in a preclinical migraine model [77]. In turn, pharmacological elevation of endogenous levels of AEA and 2-AG was found to alleviate cephalic pain hypersensitivity in the migraine models [12, 74, 78]. Thus, the reduction of 2-AG in the TGVS might lead to the increased periorbital allodynia. In this study, we found that 2-AG levels were decreased approximately 20 to 40% in TG, medulla and PAG of the TBI animals, although statistical significance was shown only in the medulla. Those findings suggest that attenuation of 2-AG mediated signaling in the trigeminal system and the key region of inhibitory pain modulation might contribute to the development of PTH, and furthermore, treatment with MJN110 to elevate the 2-AG levels could ameliorate pain initiation and transmission in the trigeminal pain pathway. This notion is also supported by our findings that the reduced production of 2-AG by co-administration of the DAGL inhibitor DO34 reversed the antinociceptive and anti-inflammatory effects of MJN110.

## Conclusions

Augmentation of the endogenous 2-AG levels by inhibiting MAGL could attenuate periorbital allodynia, glial cells accumulation, and the expression of CGRP in the TBI mouse trigeminal system. This study demonstrated the potential of endocannabinoid system modulation in the treatment of PTH, although the underlying mechanisms remain to be elucidated.

## Data Availability

Data is provided within the manuscript.

## Abbreviations

AEA: Anandamide
2-AG: 2-arachidonoyl glycerol
CB1R: Cannabinoid type 1 receptor
CB2R: Cannabinoid type 2 receptor
CGRP: Calcitonin gene-related peptide
CHIMERA: Closed head impact model of engineered rotational acceleration
CLR: Calcitonin receptor-like receptor
CSF: Cerebral spinal fluid
DAGL: Diacylglycerol lipase
ECS: Endocannabinoid system
FAAH: Fatty acid amide hydrolase
GPRs: G protein coupled receptors
5-HT1: Serotonin
IHC: Immunohistochemistry
LC/MS: Liquid chromatography/mass spectrometry
MAGL: Monoacylglycerol lipase
rmTBI: Repetitive mild traumatic brain injury
NAPE-PLD: N-acyl phosphatidylethanolamine phospholipase D
NSAIDs: Non-steroidal anti-inflammatory drugs
PACAP: Pituitary adenylate cyclase-activated polypeptide
PAG: Periaqueductal gray
PIP2: Phosphatidylinositol 4,5-bisphophate
PPARs: Peroxisome proliferator-activated receptors
PTH: Posttraumatic headache
RAMP1: Receptor activity modifying protein 1
TBI: Traumatic brain injury
TG: Trigeminal ganglion
TGVS: Trigeminovascular system
TRPs: Transient receptor potentials
TRPV1: Transient receptor potential vanilloid receptor 1
TNC: Trigeminal nucleus caudalis
VGCC: Voltage gated calcium channel
